# Assessment of the Public Health Risks and Impact of a Tornado in Funing, China, 23 June 2016: A Retrospective Analysis

**DOI:** 10.3390/ijerph14101201

**Published:** 2017-10-10

**Authors:** Kaiwen Wang, Shuang Zhong, Xiaoye Wang, Zhe Wang, Lianping Yang, Qiong Wang, Suhan Wang, Rongrong Sheng, Rui Ma, Shao Lin, Wenyu Liu, Rongqiang Zu, Cunrui Huang

**Affiliations:** 1 School of Public Health, Sun Yat-sen University, No. 74, 2nd Yat-Sen Road, Yuexiu District, Guangzhou 510080, China; wangkw6@mail2.sysu.edu.cn (K.W.); yanglp7@mail.sysu.edu.cn (L.Y.); wangqiong@mail.sysu.edu.cn (Q.W.); wangsuh@mail.sysu.edu.cn (S.W.); shengrr@mail2.sysu.edu.cn (R.S.); marui6@mail2.sysu.edu.cn (R.M.); 2 School of Government, Sun Yat-sen University, Guangzhou 510275, China; amigo-008@163.com; 3 Public Health Emergency Center, Chinese Center for Disease Control and Prevention, Beijing 102206, China; wangxy2@chinacdc.cn (X.W.); wangzhe@chinacdc.cn (Z.W.); 4 School of Public Health, State University of New York at Albany, Albany, NY 12222, USA; slin@albany.edu; 5 Funing County’s Center for Disease Control and Prevention, Yancheng 224400, China; mkaren614@hotmail.com; 6 Department for Acute Infectious Disease Control, Jiangsu Provincial Center for Disease Control and Prevention, 172 Jiangsu Road, Gulou District, Nanjing 210009, China

**Keywords:** tornado, public health impact, natural disaster, death and injury, infectious disease

## Abstract

(1) Background: Tornadoes are one of the deadliest disasters but their health impacts in China are poorly investigated. This study aimed to assess the public health risks and impact of an EF-4 tornado outbreak in Funing, China; (2) Methods: A retrospective analysis on the characteristics of tornado-related deaths and injuries was conducted based on the database from the Funing’s Center for Disease Control and Prevention (CDC) and Funing People’s Hospital. A change-point time-series analysis of weekly incidence for the period January 2010 to September 2016 was used to identify sensitive infectious diseases to the tornado; (3) Results: The 75 to 84 years old group was at the highest risk of both death (RR = 82.16; 95% CIs = 19.66, 343.33) and injury (RR = 31.80; 95% CI = 17.26, 58.61), and females were at 53% higher risk of death than males (RR = 1.53; 95% CIs = 1.02, 2.29). Of the 337 injuries, 274 injuries (81%) were minor. Most deaths occurred indoors (87%) and the head (74%) was the most frequent site of trauma during the tornado. Five diseases showed downward change-points; (4) Conclusions: The experience of the Funing tornado underscores the relative danger of being indoors during a tornado and is successful in avoiding epidemics post-tornado. Current international safety guidelines need modification when generalized to China.

## 1. Introduction

Tornadoes, violent rotating storms of a small diameter, are considered to be one of the top natural disaster killers and a major public health concern [1]. They are capable of completely destroying well-made structures, uprooting trees, and rolling up debris, usually causing mass casualties and property losses through the strong wind force and accompanied storms, hailstones, or lightning. Globally, tornadoes occur predominantly in the United States, killing 60–65 people and injuring 1500 people annually [2,3]. Although the majority of tornadoes occur in the U.S., these weather-related disasters also happen in Central and South Asia [4]. In China, 2201 tornadoes occurred between 1984 and 2013, averaging 73 times a year, killing 2000 people and injuring 30,000 people in total [5]. However, the public health risks and impact of tornadoes in these settings are not well investigated.

Previous studies on the public health-related impact of tornadoes focused mostly on death and injury in the U.S. [6,7,8,9,10,11,12]. Known risk factors include being older than 60 years, not seeking shelters, and being in mobile homes, vehicles, or outdoors. Epidemiological evidence showed that tropical cyclones including hurricanes, typhoons, and accompanied tornadoes can increase the likelihood of infectious diseases because of the disruption of public-health infrastructure, limited health services, damage to sanitation, a crowded displaced population, and other environmental change [13]. Although more studies have been conducted internationally, the conclusions are still inconsistent and current recommendations may be not suitable for other countries because of the climatic, topographic, demographic, and socioeconomic differences. In China, even though historical tornadoes have already taken many lives, surprisingly, there is no published study assessing the public health risks and impact of tornadoes.

To fill the gap, we aimed to conduct a case study of the tornado occurred in Funing, 23 June 2016, considered to be the worst tornado in China since 1977, to investigate the risk factors of tornado-related casualties and identify whether there were any infectious disease epidemics because of tornadoes.

## 2. Materials and Methods

### 2.1. Study Area

**Figure 1:**
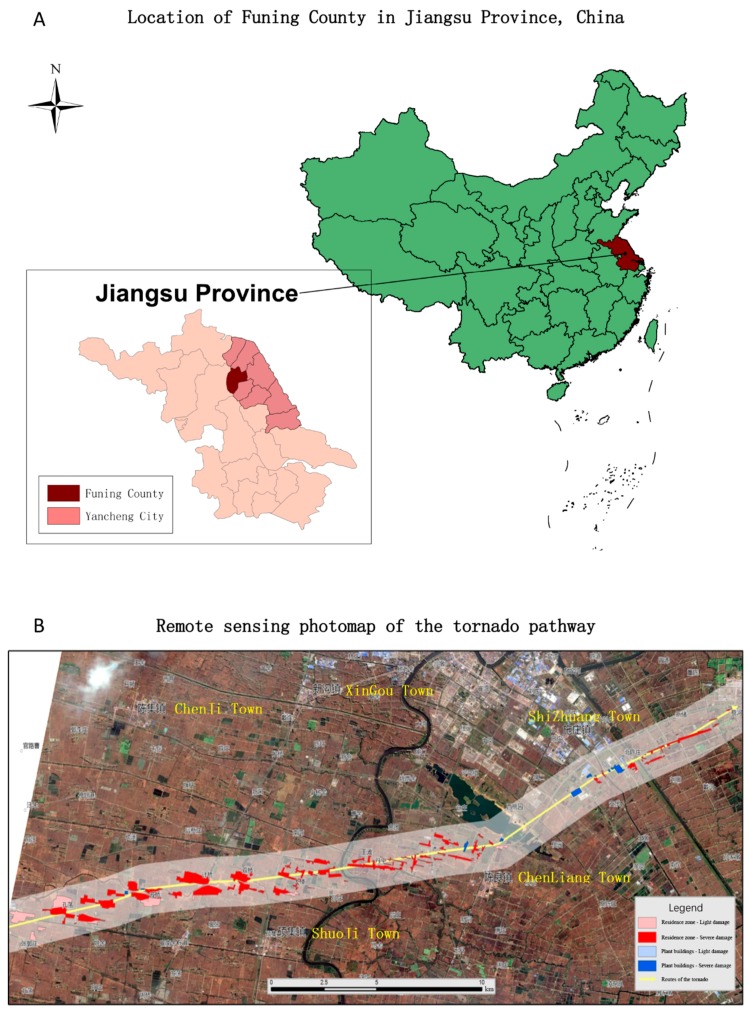
Location and pathway of the Funing tornado, 23 June 2016. (**A**) Location of Funing county in Jiangsu Province, China; (**B**) Remote sensing photomap of the tornado pathway.

Funing county, part of Yancheng city, is located in the north of inland Jiangsu Province (Figure 1A), which has experienced most tornadoes in China (21.4 times annually). It contains 13 towns with a total population of 1.1 million. Situated in a transitional climate zone, the monsoon characteristic is significant, with abundant rainfall and sunlight along with frequent extreme weather events such as floods, droughts, and extreme heat or cold spells.

During the afternoon of 23 June 2016, an EF-4 tornado (enhanced Fujita scale categories from zero to five represent increasing degrees of tornado-related damage [14]) accompanied by heavy hail and thunderstorms struck rural areas of Funing county, causing a wide range of destruction along the tornado path (Figure 1B). Approximately 4.5 km^2^ of residential areas were severely damaged, and 99 deaths and mass injuries were caused among eight towns in Funing (Banhu, Chenliang, Fucheng, Guoshu, Shizhuang, Shuoji, Wutan, and Xingou). This was the second-deadliest tornado in China, surpassing the 1966 event that resulted in 87 fatalities, also in Jiangsu Province.

The damaged area was rural and far away from hospitals, mostly consisting of farmlands and low-rise brick houses. Drinking water of households in Funing were all covered by tap water. Disaster preparedness and risk reduction system targeted to tornadoes is lacking.

### 2.2. Data

Population database was from the National Population Census data (2015) provided by the National Statistics Bureau of China. Fatality records of Funing county in 2016 were obtained from National Diseases Surveillance Points System (DSP), collected from the Funing County’s Centers for Disease Control and Prevention (CDC) in October 2016. The records capture the victims’ demographic information including age, sex, education, marriage, occupation, and the circumstances including date, location, and cause of death. The cause of death was classified using the International Classification of Diseases, 10th Revision code (ICD-10), along with the detailed description in fatality records. Location of decedents during tornado included indoors, outdoors, and vehicle. Location of deaths included on scene, in hospital, and on the way. Data on injuries were collected from Funing People’s Hospital, where most victims were taken on the day of the tornado. Case reports of injured people capture data on sex, age, and severity of injuries which has been classified as minor or severe by diagnosis.

Surveillance data on notifiable infectious diseases from January (1st week) 2010 to September (40th week) 2016 in Funing county were obtained from the National Notifiable Disease Surveillance System (NDSS). Our analysis included incidence of nine infectious diseases including unclassified hepatitis, tuberculosis, influenza, parotitis, other infectious diarrhea, hand-foot-mouth disease (HFMD), chickenpox, scrub typhus, and hepatitis B (sexually transmitted diseases and diseases with maximum weekly counts under five were excluded). Weekly counts of each disease before and after the onset of the tornado (26th week in 2016) were analyzed to identify if there were significant change-points. 

### 2.3. Statistical Analysis

This study was conceptualized as a retrospective analysis. We used 2015 Census data for age and sex from 8 affected towns in Funing county as denominators to determine age- and sex-specific rates of tornado-related death and injury. We also used this population to calculate the risk ratio (RR) and 95% confidence intervals (CIs) of tornado-related death and injury for the demographic variables available in the 2015 Census compared to the reference groups (35–44 years old for age group and males for sex group). Time series analysis was used to perform trends of each infectious disease pre- and post-tornado by week. To identify sensitive diseases, we used change-point analysis (CPA) to detect significant shift in infectious diseases’ trends. We assumed that a disease with a significant change-point immediately after the tornado (26th week, 2016) in time series (1st week, 2010–40th week, 2016) can be considered as a sensitive disease. Data were analyzed using R software version 3.3.2 (R Foundation for Statistical Computing, Vienna, Austria).

### 2.4. Ethical Approval

This study was approved by the medical ethics committee of School of Public Health, Sun Yat-sen University (L2016-012). Data used in the study were anonymous and individual patient consent was not required.

### 2.5. Availability of Data and Materials

The data that support the findings of this study are available from Funing County’s Centers for Disease Control and Prevention and Funing People’s Hospital but restrictions apply to the availability of these data, which were used under license for the current study, and so are not publicly available. Data are however available from the authors upon reasonable request and with permission of Funing County’s Centers for Disease Control and Prevention and Funing People’s Hospital.

## 3. Results

**Figure 2:**
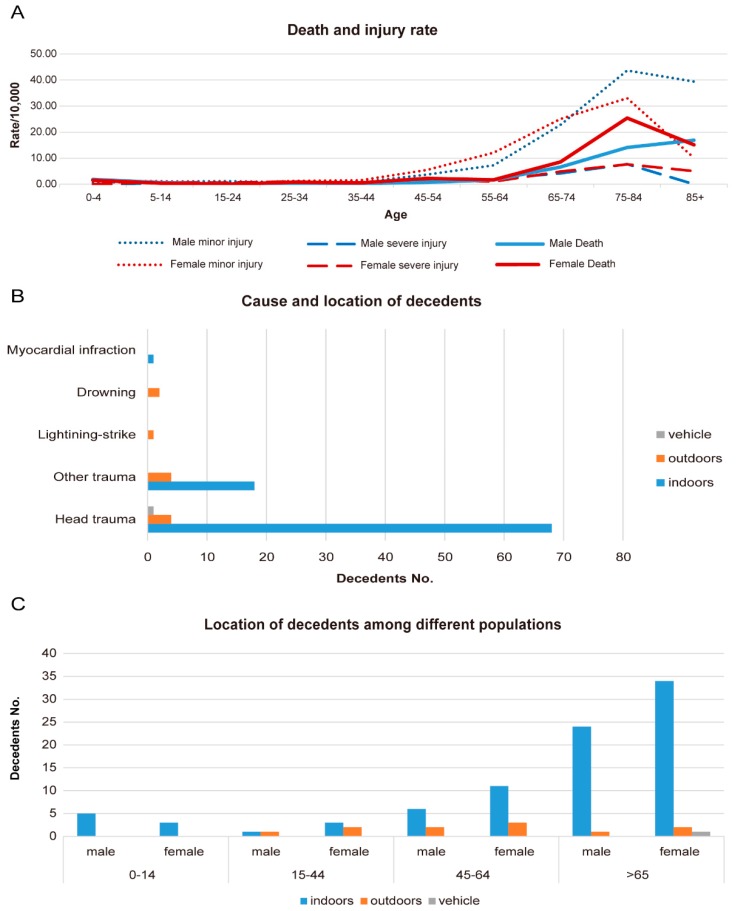
Characteristics of deaths and injuries of the tornado in Funing, 23 June 2016. (**A**) Death and injury rate among males and females; (**B**) cause and location of decedents; (**C**) location of decedents among different age and gender groups.

We identified 99 deaths in total directly attributable to the Funing tornado on 23 June 2016. Table 1 showed the demographics and circumstances of the decedents. The mean age of decedents was 63 years, ranged from 1 to 89 years. Majority of decedents were adults over 45 years old (86%), females (60%), married (68%), farmers (89%), and those with a level of education below junior middle school (98%). Children younger than 5 years old were about 7 times more likely to die (RR = 7.13; 95% CI = 1.38, 36.74). The risk of death increased with age among adults (Figure 2A), reaching statistical significance among decedents older than 45 years old, and the 75 to 84 years old group was at the highest risk of death (RR = 82.16; 95% CI = 19.66, 343.33). Females were at approximately 53% higher tornado-related death risk than males (RR = 1.53; 95% CI = 1.02, 2.29).

Among all the decedents, 79 people (79.8%) died at the scene, 18 people died in hospital, and two died on the way to hospital (Table 1). Most deaths were caused by head trauma (*n* = 73; 73.7%) that occurred mostly indoors (Figure 2B), except for one death was caused by myocardial infarction after the victim had been shocked by his wife’s death, two were drowned, and the other death was due to lightning-strike. More people were killed indoors (*n* = 87; 87.9%) than outdoors (*n* = 11; 11.1%) or in a vehicle (*n* = 1; 1.0%) when the tornado struck them (Table 1). The majority of indoor decedents were the elderly older than 65 years old, while decedents who died outdoors were mostly young and middle-age adults. Child decedents younger than 14 years old were found to have all died at home (Figure 2C).

Table 2 showed demographics of injuries. Of the 337 injuries we obtained, 274 injuries (81%) were minor. Within the 8 tornado-affected towns of Funing, rates for minor injury and severe injury were 5.39 and 1.24 per 10,000 population, respectively. In adults aged over 45 years, both minor and severe injury rates increased with age similarly to the pattern of death rates, but among younger age groups (under 45 years), we found no consistent pattern (Figure 2A). Severe injury rates were always lower than death and minor injury rates in adults, and the elderly aged between 75 and 84 were at the highest risk of both injury (both minor and severe) and death. However, there was no significant difference in risk of injuries among males and females (Table 2).

**Figure 3:**
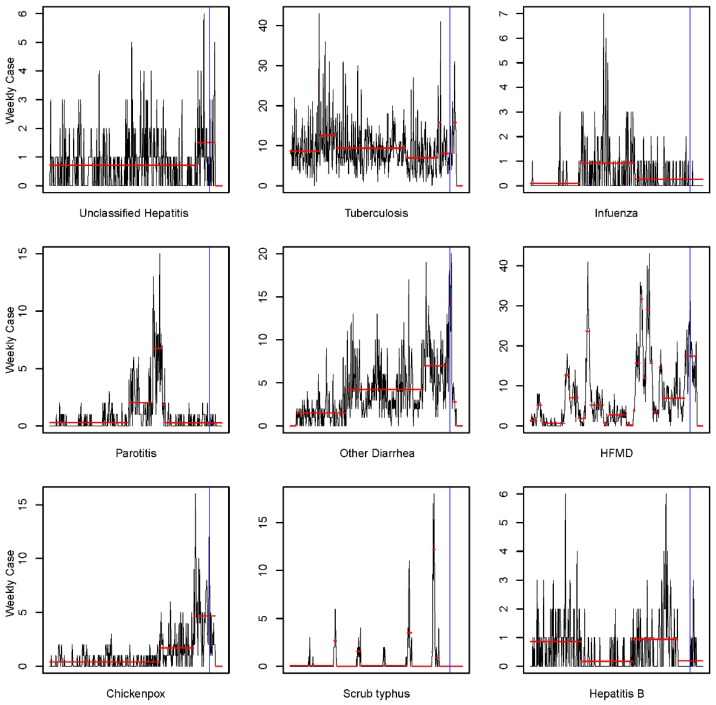
Change-points of notifiable infectious diseases in Funing from January 2010 to September 2016. Results show the time series (1st week, 2010–40th week, 2016) of weekly cases on nine notable infectious diseases. Blue line represents the onset of tornado.

As shown in Figure 3, none of the notifiable diseases showed an increasing trend after this tornado. Downward change-points appeared after the onset of the tornado among five infectious diseases including unclassified hepatitis, tuberculosis, other diarrhea, HFMD, and chickenpox. Notably, the change-points after the tornado in other diarrhea and unclassified hepatitis are the first and only downward change-points in the entire six-years-time series. The bottom of unclassified hepatitis was marked on the 38th week in 2016 (12 weeks after tornado), and counts of other diarrhea showed the first significant decreasing point on the 30th week in 2016 (4 weeks after tornado).

## 4. Discussion

Our study found that the EF-4 tornado in Funing on 23 June 2016 caused mass casualties mainly in children, females, and the elderly, and most decedents died indoors because of head trauma. There was no clear evidence of tornado-related increase in infectious diseases in this event. To the best of our knowledge, this is the first health risk analysis of tornadoes in China. Review of the associated factors in this tornado may provide insights into policy recommendations.

In this event, children and older adults were at significantly greater risk of both tornado-related death and injury, which is consistent with previous studies in the U.S. [8,9], presumably as a result of preexistent medical illnesses, poor mobility, decreased ability to respond rapidly to tornado warnings, and greater susceptibility to injury. As nearly 90% decedents in Funing tornado were farmers, it is likely that most children, females, and aged people may have stayed indoors (which was found to be the most dangerous location in our study) at the time of tornado (2:30 p.m.), while young adults, especially males, were more likely to have been working outdoors in open areas. Similar age and gender patterns in the victims were observed in Sichuan earthquake in 2008 [15]. This finding may also reflect the socioeconomic environments in rural China where adults at working age may have migrated to urban settings for better job opportunities, leaving behind a population in which older people, women, and children are over-represented. Current policy can focus attention on the left-behind population and encourage families to take extra precaution to warn, check on, and assist these vulnerable people in seeking shelter or in evacuating the area.

Numerous studies [6,7,8,16] have identified indoors as safe locations during tornadoes, while our results are contrary to these findings. In the U.S., residents are always encouraged to remain indoors during tornadoes, and storm shelters, basements, or well-supported interior rooms (closets, bathrooms, or hallways) are readily available for protection. In the affected area of Funing in China, almost all buildings were traditional mud brick constructions, which were totally destroyed into ruins, causing nearly 90% of the decedents that died at home. Possible interpretation also includes affected inhabitants taking siesta inside the houses without any alertness at the occurring time of the tornado. Given that we do not have data on the total number of indoor and outdoor residents in the exposed areas at the time of the tornado, we were unable to estimate risk ratios. However, this event was significantly more severe than previous tornadoes; therefore, brick houses may still be documented as a dangerous location during an EF-4 tornado in rural China. Considerable investment in reconstructing and shelter-building is critical in preventing tornado-related death in tornado-prone areas. It may also suggest that in the most violent (EF-4 and EF-5) tornadoes, even location historically considered safe may not guarantee survival. 

Moreover, almost all of the decedents in Funing tornado sustained head injuries. To protect the high-risk trauma site, helmet-use was already found to be an effective method to prevent head-injuries during tornado in the U.S. [10] However, this method has still not been widely adopted nor been educated. Compared to reconstructing solid buildings, encouraging residents wearing helmets or blankets on the head during tornadoes may be a cost-effective and convenient way to avoid high-risk deaths caused by head trauma, especially in areas without shelters.

Another finding is that minor injuries were far more frequent than severe injuries in this event, similar to a previous study [17], suggesting those with minor injuries may have more opportunities to survive than those with severe injuries. Severe injuries were usually fatal injuries with massive haemorrhage or trauma, thus requiring immediate emergency medicine or surgery. However, the local medical responders cannot deal with all the severe injuries immediately and effectively due to the limitations in the shortage of first-aid rescue resources and damaged transferring conditions, thus severely delaying the best window of rescue time. The fact that the majority of decedents died at the scene may also support the proposition that investments in heightening emergency medicine will be critical to preventing a number of tornado-related deaths [18].

The Chinese government should be commended for its successful avoidance of epidemics, as we found no outbreaks of infectious disease after this tornado. This phenomenon may result from stringent regulations on post-disaster infectious disease control in China. Authorities always switched their focus to epidemics to prevent a secondary health disaster immediately after the disposal of casualties. Local institutions have to take actions routinely to ensure the safety and sanitation of water and food to prevent gastrointestinal diseases. Disinfection seemed to be well done as vector-borne diseases were found sporadically with no indication of outbreaks, even though it was summer when the tornado occurred. To our knowledge, the child immunization coverage rate in Funing (98%) was far above the average level of whole of China (around 90%). This may account for the stabilization of measles, mumps, tuberculosis, and hepatitis B, which can be successfully prevented from vaccination.

Natural disasters are best known for their sudden health impacts: death, injury, and epidemics, as we mentioned above. However, momentum in relief work and the reinforcement of attention payed to chronic disease and psychological problems must be maintained to avoid a secondary disaster of non-communicable diseases. Among the decedents of the Funing tornado, one old man died from myocardial infarction due to the shock over the death of his wife, which may suggest that chronic illness can be exacerbated by disasters, especially for older persons who are more susceptible to adverse effects of both psychological and physical stress caused by disasters because of higher rates of chronic illnesses [19]. Myocardial infarction, stroke, and pneumonia were shown to increase post-tornado in previous events [20,21]. Sometimes, survivors without immediate surgical needs but with potential life-threatening medical conditions (e.g., cardiovascular or respiratory diseases) may not always receive best management, despite the availability of drugs and resources. In addition to the challenge of rebuilding their socioeconomic support systems, they face not only the critical need for tornado-related trauma, but also the continuing need for clinical management of complex chronic conditions. However, past experience in the Japanese and Pakistani earthquakes, as well as in Hurricane Katrina [22,23,24], documented that chronic medical needs post-disaster were often inadequately managed and can result in increased rates of complication and indirect morbidity after a disaster. 

Improvement in severe storm forecasting technologies, development of tornado warning systems, growing public awareness, and improved house construction have helped lower the death toll of tornado over years in the U.S. [25]; however, China still has a formidable task ahead. Our study suggests the following recommendations, that may reduce tornado-related casualty in China: (1) improvements in building structure in regions at risk of tornadoes; (2) construction of tornado-resistant shelters or basements; and (3) institution of public education programs that teach appropriate protective behaviors during tornadoes, such as escaping to open space (if shelters are unavailable), and wearing helmets or blankets on the head. In addition, we reiterate that targeted precautions should be taken to warn vulnerable groups like children, females, and the elderly. Social media was found to be a potential disaster risk reduction tool during communications and shelter-seeking in tornado events [26,27,28,29,30]. In China, Webchat is now very popular and is used by 0.77 billion people, so future research may develop the competency of social media to assist in disaster management.

The boom of a research field often follows a catastrophe event. For instance, in China, a surge in attention being paid to epidemics followed the outbreak of SARS (Severe Acute Respiratory Syndromes) in 2003, and the focus on PTSD increased during the aftermath of Wenchuan earthquake in 2008. Tornadoes have played a negligible part in Chinese disaster preparedness efforts for many years; however, the intense windstorms involving tornadoes are expected to increase and become more extreme due to climate change in the future [31]. Disasters like tornadoes cannot be viewed only as mass-casualty incidents but also a public health challenge that needs long-term risk reduction and management. As a large and diverse country, other tornado-prone places in China like Anhui and Guangdong provinces may have different features from Jiangsu because of differences in terrain, climate, and sociodemographic conditions. Caution must be used in generalizing findings from this event, which was dominated by an extraordinarily powerful tornado (EF-4), to less powerful tornadoes that are more sudden and transient. Meteorological departments and public health officials are calling for more robust evidence to develop a flexible list of recommendations that can account for differences in magnitude, location, and warning time, which can be provided through more multicenter studies in the future. This paper will be an opening of tornado-related health research in China and arouse more concerns in this field to reduce future tornado-related health loss.

Furthermore, important impacts on health will include changes in the frequency and intensity of extreme weather events [32]. Climate change projections have indicated warmer climate may lead to more frequent, severe storms, which may lead to lager public health burden [33,34]. Better management, mitigation, and deployment of storm warnings could save more lives in the future, and greater emphasis is needed on understanding the health impacts of, and supporting vulnerable communities to effectively prepare for, respond to, and recover from, such extreme events [35,36].

There are several limitations to this study. Firstly, we only obtained injury cases from Funing People’s Hospital. Injured people who were sent to other community hospitals or village doctors were dispersed and we were unable to locate them. Secondly, most residents in the affected areas cannot understand or speak Mandarin; thus, field survey was hard to conduct due to the language barrier. Surveys on survivals may be conducted with the help of local staff in the future to obtain more detailed information. Lastly, only deaths, injury, and infectious disease data were contained in this study. Morbidity (especially non-communicable diseases and mental disorders) data could not obtain. This issue will be addressed in our ongoing study on long-term health impact of tornadoes, which is designed to collect more hospitalization data.

## 5. Conclusions

To the best of our knowledge, this is the first study that documents the public health risks and impact of tornadoes in China. The experience of the Funing tornado of 23 June 2016 underscores the relative danger of being indoors during a tornado. It also raises the importance that some international tornado safety guidelines need modification when generalized to China. Considering the improved tornado warning technology, escaping outdoors may be a viable alternative if no shelter is immediately available. Otherwise, wearing helmets or blankets may offer greater protection for residents who were limited indoors during a tornado, especially in children, the elderly, and females. The Chinese government should be commended for its successful avoidance of epidemics through strict and normative surveillance and disinfection, which may be an example for other developing countries; however, public health institutions must still maintain momentum in their emergency rescue and relief work. Future research is needed on the long-term burden of tornadoes, such as non-communicable diseases and mental health.

## Figures and Tables

**Table 1:** Demographics and circumstances of decedents in the Funing tornado, 23 June 2016.

Variables	No. (%)	Population	Rate ^a^	RR (95% CI)
**Age**				
0–4	5 (5.1)	29,126	1.72	7.13 (1.38, 36.74) *
5–14	3 (3.0)	55,729	0.54	2.23 (0.37, 13.38)
15–24	0 (0.0)	68,725	0.00	0.00
25–34	4 (4.0)	78,797	0.51	2.11 (0.39, 11.51)
35–44	2 (2.0)	83,031	0.24	1.00 (Ref.)
45–54	12 (12.1)	80,585	1.49	6.18 (1.38, 27.62) *
55–64	10 (10.1)	60,029	1.67	6.92 (1.52, 31.56) *
65–74	26 (26.3)	33,054	7.87	32.66 (7.75, 137.59) *
75–84	31 (31.3)	15,664	19.79	82.16 (19.66, 343.33) *
85+	6 (6.1)	3759	15.96	66.27 (13.37, 328.43) *
**Sex**				
Male	40 (40.4)	259,087	1.54	1.00 (Ref.)
Female	59 (59.6)	249,412	2.37	1.53 (1.02, 2.29) *
**Marriage**				
Married	67 (67.7)			
Widowed	20 (20.2)			
Single	10 (10.1)			
Divorce	2 (2.0)			
**Education**				
<Junior middle school	97 (98.0)			
>Senior high school	2 (2.0)			
**Occupation**				
Farmer	88 (88.9)			
Child	6 (6.1)			
Student	4 (4.0)			
Worker	1 (1.0)			
**Cause of death**				
Trauma	95 (96.0)			
Head	73 (73.7)			
Other sites	22 (22.3)			
Myocardial infarction	1 (1.0)			
Drowning	2 (2.0)			
Lightning-strike	1 (1.0)			
**Location during the tornado**				
Indoors	87 (87.9)			
Outdoors	11 (11.1)			
Vehicle	1 (1.0)			
**Location of death**				
At the scene	79 (79.8)			
In hospital	18 (18.2)			
On the way to hospital	2 (2.0)			
**Total**	99			

Note: CI = confidence interval; RR = risk ratio. ^a^ Rate per 10,000 populations in 8 affected towns of Funing County (Banhu, Chenliang, Fucheng, Guoshu, Shizhuang, Shuoji, Wutan, Xingou); * Indicates statistically significant data.

**Table 2:** Demographics of injuries in the Funing tornado, 23 June 2016.

	Minor Injury			Severe Injury			Total Injury		
	No.	Rate ^a^	RR (95% CI)	No.	Rate ^a^	RR (95% CI)	No.	Rate ^a^	RR (95% CI)
**Age**									
0–4	3	1.03	0.95 (0.26, 3.51)	0	0.00	0	3	1.03	0.71 (0.20, 2.53)
5–14	6	1.08	0.99 (0.35, 2.79)	3	0.54	1.49 (0.30, 7.38)	9	1.61	1.12 (0.47, 2.65)
15–24	5	0.73	0.67 (0.22, 2.00)	3	0.44	1.21 (0.24, 5.99)	8	1.16	0.81 (0.33, 1.97)
25–34	8	1.02	0.94 (0.36, 2.43)	7	0.89	2.46 (0.64, 9.51)	15	1.90	1.32 (0.62, 2.81)
35–44	9	1.08	1.00 (Ref.)	3	0.36	1.00 (Ref.)	12	1.45	1.00 (Ref.)
45–54	37	4.59	4.24 (2.04, 8.78) *	10	1.24	3.43 (0.95, 12.48)	47	5.83	4.04 (2.14, 7.61) *
55–64	58	9.66	8.91 (4.42, 17.99) *	9	1.50	4.15 (1.12, 15.33) *	67	11.16	7.72 (4.18, 14.28) *
65–74	79	23.90	22.05 (11.06, 43.95) *	15	4.54	12.56 (3.64, 43.39) *	94	28.44	19.68 (10.79, 35.89) *
75–84	60	38.30	35.34 (17.53, 71.22) *	12	7.66	21.20 (5.98, 75.14) *	72	45.97	31.80 (17.26, 58.61) *
85+	9	23.94	22.09 (8.76, 55.68) *	1	2.66	7.36 (0.77, 70.80)	10	26.60	18.41 (7.95, 42.63) *
**Sex**									
Male	130	5.02	1.00 (Ref.)	28	1.08	1.00 (Ref.)	158	6.10	1.00 (Ref.)
Female	144	5.77	1.15 (0.91, 1.46)	35	1.40	1.30 (0.79, 2.13)	179	7.18	1.18 (0.95, 1.46)
**Total**	274	5.39		63	1.24		337	6.63	

Note: CI = confidence interval; RR = risk ratio. ^a^ Rate per 10,000 populations in 8 affected towns of Funing County (Banhu, Chenliang, Fucheng, Guoshu, Shizhuang, Shuoji, Wutan, Xingou); * Indicates statistically significant data.
